# Cell-Penetrating Peptides to Enhance Delivery of Oligonucleotide-Based Therapeutics

**DOI:** 10.3390/biomedicines6020051

**Published:** 2018-05-05

**Authors:** Graham McClorey, Subhashis Banerjee

**Affiliations:** Department of Physiology, Anatomy and Genetics, University of Oxford, Oxford OX1 3QX, UK; subhashis.banerjee@dpag.ox.ac.uk

**Keywords:** cell-penetrating peptides, antisense oligonucleotides, delivery

## Abstract

The promise of nucleic acid based oligonucleotides as effective genetic therapies has been held back by their low bioavailability and poor cellular uptake to target tissues upon systemic administration. One such strategy to improve upon delivery is the use of short cell-penetrating peptides (CPPs) that can be either directly attached to their cargo through covalent linkages or through the formation of noncovalent nanoparticle complexes that can facilitate cellular uptake. In this review, we will highlight recent proof-of-principle studies that have utilized both of these strategies to improve nucleic acid delivery and discuss the prospects for translation of this approach for clinical application.

## 1. Introduction

This century is being heralded as the era of genomic medicine. With the mapping of the human genome sequence, advancement in the field of genetic engineering has transformed our understanding of how genes underpin biological processes in health and disease states and has principally brought the whole genome amenable to sequence-specific therapeutic intervention via nucleic acid based molecules. The concept of “personalized medicine” has thus become a reality and various gene-manipulative technologies such as antisense methodologies have demonstrated “proof-of-principle” for treatment of rare genetic diseases. However, these antisense biomolecules—namely, small interfering RNAs (siRNAs), classical antisense oligonucleotides (ASOs), splice-switching ASOs (SSOs), microRNAs (miRNAs), and anti-miRNAs (antagomirs)—are generally high molecular weight, highly charged entities and thus cannot easily traverse the cell membrane. This is especially the case for neurodegenerative disorders where the tightly-regulated blood-brain barrier (BBB) prevents uptake of most pharmaceuticals. This hurdle of low bioavailability and poor cellular uptake of biologically active nucleic acids means there is an urgent need to address this in the field of drug delivery. Cell-penetrating peptides (CPPs, also known as protein transduction domains) could be one of the solutions to this problem. In comparison to the extensively studied traditional delivery systems like liposomes, viral vectors, or polymeric cation-based systems (polyplexes) [1,2], CPPs are unique in that they are short (fewer than 30 amino acids) cationic and/or amphipathic peptides that translocate small drugs/cargo across cell membranes [3,4]. Many of the early identified CPPs derive from peptide sequences found in naturally occurring protein elements that exhibited inherent translocating properties. Some of the most important of these for subsequent CPP iterations include: the transactivator of transcription from HIV (HIV-Tat) [5]; Penetratin-1, which is derived from the homeodomain of Antennapedia, a *Drosophila* transcription factor [6]; Transportan, a chimeric peptide derived from galanin and the wasp-venom peptide toxin mastoparan [7]; and cationic polyarginine and polylysine sequences such as Arg8 [8]. Several types of cargoes, for example, proteins, nucleic acid based macromolecules such as siRNA, plasmid DNA, ASOs, and small drug molecules, can be transported by CPPs to overcome the natural cellular biological barriers. This has led to increased interest in the utilization of CPPs as delivery tools of genetic modifiers, with some of the greatest promise demonstrated for nucleic acid delivery to organs of the body with inherently poor uptake by naked delivery. Examples of some of these CPPs and their applications are listed in Table 1 and this review intends to highlight the progress of CPP development for oligonucleotide delivery that could expand the repertoire of novel “druggable” targets.

## 2. Direct CPP Conjugation for Nucleic Acid Delivery

**Figure 1:**
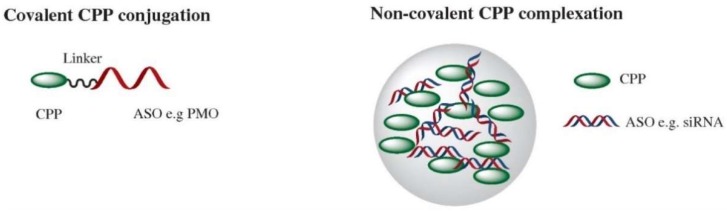
Cell-penetrating peptides (CPPs) can be conjugated to their ASO cargo through direct covalent conjugation through a linker, most typically exemplified by cationic or amphipathic CPP conjugation to a neutral-charge oligonucleotide such as phosphorodiamidate morpholino (PMO). Noncovalent conjugation occurs through electrostatic and hydrophobic interactions between the CPP and ASO, exemplified by siRNA here, to form nanoparticle like complexes.

Within the context of CPP-mediated delivery, effector nucleic acids can either be directly conjugated to the CPP or noncovalently complexed, typically forming nanoparticle structures (Figure 1). Covalent conjugation occurs with a defined structure and stoichiometry between predominantly charge-neutral oligonucleotide chemistries, such as peptide nucleic acids (PNA) and phosphorodiamidate morpholino (PMO), and cationic CPPs. CPPs (10–16 residues long) join covalently to the cargo antisense nucleic acid and then traverse the biological membranes through various peptide-mediated uptake mechanisms. Neutral-charge backbone chemistries are an ideal cargo for charged CPPs as they avoid problems of electrostatic interaction and subsequent aggregation that can occur with anionic chemistries. One of the earliest studies to demonstrate the potential of direct CPP conjugation was the addition of four lysines to a PNA sequence that had enhanced splice-switching activity in a GFP reporter mouse model compared to naked PMO [33]. The ability of CPPs to enhance PNA activity was further investigated across a range of CPP classes, including Transportan, oligo-arginine, pTat, Penetratin, KFF, SynB3, and NLS, in luciferase-based splice-switching assays, with transportan 10 having the highest efficacy [34]. A key finding of this study was that presence of serum led to inhibition of activity of a subset of CPPs and this may partly explain the relatively few successful in vivo studies with CPP-PNAs. Nevertheless, CPP-PNAs have been utilized in preclinical studies targeting c-myc for severe combined immunodeficiency [35] and as a proof of concept as potent in vivo inhibitors of miRNA upregulation following lipopolysaccharide induction [36]. One of the most promising neutral-charge chemistries are PMOs that consist of a morpholine ring in place of ribose and phosphorodiamidate linkages in place of phosphodiester [37]. These compounds act primarily through steric blockage of complementary RNA sequences to either inhibit protein translation or to modify pre-mRNA splicing. As PMOs do not efficiently enter cells on their own, the strategy of CPP conjugation has been utilized to improve cellular uptake and enhance their antisense activity [38]. Conjugation of CPP to PMO uses several methods, including maleimide linkage, disulfide linkage, click chemistry, or amide linkage [39] and enhances the PMO pharmacokinetic profile, biodistribution, and stability [40]. The most promising results to date have been with arginine-rich peptides that contain aminohexanoic acid and beta-alanine spacers to improve delivery of splice-switching oligonucleotides (SSOs) [41]. For therapeutic purposes, this approach has been explored most extensively for Duchenne muscular dystrophy (DMD), which affects 1 in 3500 newborn boys and is caused primarily by out-of-frame deletions in the *DMD* gene that results in the loss of the structural muscle protein dystrophin. Absence of dystrophin causes progressive muscle degeneration, resulting in loss of ambulation and premature death due to respiratory and cardiac failure. Targeting of pre-mRNA splicing through an “exon-skipping” approach restores the mRNA reading frame around the deletion, producing an internally deleted yet still functional protein. This approach has been used successfully with naked PMO that resulted in the conditional approval of Eteplirsen, a PMO targeting exon 51 of the *DMD* gene [42]. However, despite the safe profile of this drug and evidence of dystrophin production, a limitation was the relatively low treatment efficacy, with restoration of dystrophin an average of 0.9% of normal levels following 180 weeks treatment. A need therefore remains for a more effective compound to improve dystrophin levels and thus functional benefit from this approach. The use of CPPs to improve PMO delivery is one such approach and was first demonstrated with arginine-rich B-peptide (B-PMO) that demonstrated approximately 50% wild-type dystrophin levels following a single 25 mg/kg dose [9] concurrent with partial restoration in cardiac function measures following dobutamine-mediated stress induction, and improved muscle function [10] following intravenous systemic administration into the *mdx* mouse model of DMD. This compares extremely favorably to naked PMO, as weekly administration of PMO at 200 mg/kg for 12 weeks could only achieve 10% wild-type dystrophin levels [43]. Subsequent utilization of a fusion peptide of B-peptide with a muscle-targeting peptide that was identified through a phage display (MSP) [44] was able to improve activity 2- to 4-fold after multiple 6 mg/kg dosing, although interestingly, in a further study, the orientation of the peptide fusion in relation to PMO conjugation was shown to be an important factor in activity [12]. B-PMO has also been utilized for studies in canine models of DMD that greater recapitulate the human disease pathology and thus represent a sterner test of the ability of CPP-PMOs to effectively restore dystrophin expression. Intravenous administration of repeat low dose (4 mg/kg per ASO) B-PMO induced body-wide dystrophin restoration at approximately 5% of wild-type levels, including in the heart where amelioration of cardiac conduction abnormalities was observed after treatment [45]. This was an important proof-of-principle study to demonstrate that CPP-PMO could have success in dystrophic muscle environments that can contain additional barriers to uptake, such as fibrosis and fat deposition. B-PMO has also been utilized as a therapeutic strategy for targeting myotonic dystrophy type I, where a CTG expansion in the 3′ untranslated region of the dystrophia myotonica-protein kinase (*DMPK*) gene results in a pathogenic transcript that complexes with RNA-binding proteins such as muscleblind-like 1 (MBNL1), resulting in widespread aberrant splicing abnormalities. Systemic administration of B-PMO targeting this repeat element blocked Mbnl1 sequestration, resulting in normal nuclear distribution and subsequent correction of abnormal RNA splicing, including for chloride channel 1 gene, which is a primary contributor to myotonia [11].

Our group has more recently developed a CPP class called PNA/PMO internalization peptides (Pip) that comprises a hydrophobic core region flanked on each side by an Arg-rich domain containing aminohexanoic (X) and β-alanine (B) spacers [46]. A single 25 mg/kg dose induced 25–50% wild-type dystrophin levels in body-wide skeletal muscle similar to B-PMO, but most significantly, introduction of this core sequence conferred approximately 30% dystrophin expression in the heart, which was absent for B-PMO at the same dosing level [47]. Further iterations of core design, namely Pip6 series of peptides, demonstrated variable levels of activity that suggested a minimal core sequence length is required to maintain activity, although precise sequence was less important [14]. Furthermore, a Pip6-PMO was utilized to investigate the pharmacodynamics of this approach, including the observation that approximately 15% wild-type dystrophin protein levels are required to protect against eccentric contraction-induced muscle damage, as well as parameters such as minimal efficacious dosing levels and maintenance of effect [16] [. Other novel CPPs have been developed for muscle targeting including M12 identified through phage display performed on C2C12 myoblasts [17]. Conjugation to PMO resulted in 10–25% wild-type dystrophin levels following a single systemic administration although at dosing levels 5- to 6-fold higher than that for comparable efficacy with B and Pip CPPs.

One of the biggest challenges of the nucleic acid therapeutic field has been systemic delivery to the central nervous system (CNS), where the blood–brain barrier represents a formidable barrier even for small-molecule drugs. The potential for CPPs to deliver nucleic acid cargo was demonstrated in a study whereby systemic administration of an arginine-rich CPP conjugated to a FITC-labelled PMO cargo resulted in widely detected uptake into all areas of the brain, most notably to the cerebellum and Purkinje cells, although the functional benefit of this uptake was not reported [48]. More recently, CPP-PMOs have been explored in preclinical models of spinal muscular atrophy (SMA) whereby mutations in the *SMN1* gene results in the loss of survival motor neuron (SMN) protein and subsequent severe motor developmental delay and premature death. A paralogous gene, *SMN2*, also encodes SMN protein but only produces low levels due to a sequence variant that results in exclusion of exon 7 from ~90% of mature transcript which in turn produces a truncated, nonfunctional protein. A strategy of employing 2′-O’methoxyethyl SSOs (Nusinersen) to target an intronic silencing element to promote inclusion of exon 7 in the *SMN2* gene has recently been clinically approved, having demonstrated significant improvement in motor function and survival rates in infants [49,50]. Delivery to the CNS is essential for this approach and is achieved clinically through intrathecal administration which, whilst tolerated, places a considerable burden on patients and clinicians. Systemic administration would likely be a preferable route and may also have benefit in restoring SMN levels in tissues outside the CNS. To address this, intravenous administration of Pip6A-PMO into a severe mouse model of SMA increased mean survival drastically (~38-fold), improved neuromuscular junction morphology and rescued levels of circulating insulin like growth factor 1 at 4-fold lower doses than comparable efficacy observed for naked PMOs [15]. Notably, the severity of this mouse model necessitates delivery before postnatal day 2 to show functional benefit, where it is likely that the BBB may not be fully formed, and as such does not reflect the clinical situation for therapeutic intervention. To address this, systemic administration of Pip6A-PMO to a phenotypically unaffected adult mouse model of SMA was performed in the same study with demonstration of a 0.25- to 0.5-fold increase in corrected *SMN* transcript in brain and spinal cord, and an approximately 3-fold increase in liver and skeletal muscles. A subsequent study using the same adult mouse model explored putative brain-targeting motifs to enhance PMO delivery [18]. A branched chain peptide designed to target the apolipoprotein receptor Br-ApoE(K→A) induced a 0.25-fold increase in exon 7 inclusion in the spinal cord and to a lesser extent in the brain.

One of the limitations of positively charged CPPs is that conjugation to negatively-charged nucleic acids such as siRNAs and 2′-*O*-Me ASOs results in electrostatic interactions that can self-aggregate and potentially interfere with oligonucleotide target binding. In an attempt to address this, a 7-mer phage display was performed to identify noncharged homing peptides that would enhance delivery of 2OMePS SSO in the *mdx* mouse model [19]. Conjugation of a candidate peptide (LGAQSNF) increased exon skipping activity approximately 5–10% in skeletal and cardiac tissues following a 6-week subcutaneous administration protocol, although notably an increase in protein restoration was not observed.

As well as modulating splicing to correct a genetic defect, SSOs can be utilized for destructive exon skipping whereby induction of an out-of-frame transcript results in nonsense-mediated decay and subsequent loss of protein [51,52]. As significant knockdown of gene expression, and hence protein levels, would be required for a functional effect, current naked SSO delivery technologies are likely to be too inefficient in vivo. Thus, PMOs conjugated to B peptide were utilized in an exemplar study for targeting myostatin, a negative regulator of skeletal muscle growth and differentiation, in combination with *Dmd* exon skipping as a complementary strategy for DMD [53]. Combinatorial treatment improved some, but not all, functional measures of muscle pathology in the *mdx* model to a greater extent than dystrophin expression alone, demonstrating the potential of destructive exon skipping as an alternative to more widely used siRNA and RNaseH gene knockdown strategies [54]. This combinatorial approach has also been utilized to simultaneously target two genes using a single CPP-PMO construct. Addition of a second PMO through either “click” chemistry or a disulfide linker allowed two PMOs to be delivered by a single CPP with similar splice-switching activity as having two separate CPP-PMOs, potentially offering the advantage of reduced CPP toxicity burden [55].

CPP-ASO approaches have also been developed as antibacterial agents, (reviewed in [56]), as ASOs alone have poor capability to penetrate the bacterial cell membrane. In an exemplar study, intranasal administration of (RXR)_4_-PMO targeting *acpP* in a mouse *Acinetobacter* pneumonia model showed increased survival time and reduced pulmonary bacterial levels compared to saline controls [21].

## 3. Noncovalent CPP Delivery of Nucleic acids

Noncovalent CPP strategies depend on the formation of nanoparticle complexes due to electrostatic and/or hydrophobic interactions between anionic oligonucleotides and predominantly amphipathic peptides that consist of a hydrophilic (polar) domain and a hydrophobic (nonpolar) domain. Amphipathicity of these peptides can be conferred either due to sequence-specific ordering along the peptide chain of hydrophobic and hydrophilic residues (primary peptides) or through the conformational structure of the peptide that confers hydrophilic and hydrophobic sides, such as an α-helical or β-sheet structure (secondary peptides). One of the earliest reported primary amphipathic CPPs for ASO delivery was the MPG peptide that contains a hydrophobic domain derived from the fusion sequence of HIV gp41 and a hydrophilic domain derived from the nuclear localization sequence of SV40 T-antigen [57]. A modified version of this peptide, MPG-8, was used to deliver siRNA targeting the cell cycle regulator cyclin B1 in a xenograft tumor mouse model [22]. The molar ratio of cargo to peptide was found to be a crucial determinant of efficacy, with optimal efficacy achieved at an MPG-8/siRNA molar ratio of 20/1, which produced stable particles with diameters of 120 nm. Whilst intratumoral administration of MPG-8/siRNA complexes induced complete inhibition of tumor growth at 0.25 mg/kg, intravenous systemic injection of 0.5 mg/kg induced only a 12% decrease in tumor growth. To resolve this, MPG-8/siRNA particles were functionalized with cholesterol moieties, which are suggested to improve potency and stability through longer circulation times [58]. Addition of this moiety induced significantly increased mouse survival from 20% to 70% by day 40 compared to cholesterol-free formulation and 92% reduction in tumor growth at only 0.5 mg/kg. The same group also reported development of a secondary amphipathic peptide (CADY) that consists of aromatic tryptophan (Trp) and cationic arginine residues that adopt a helical conformation within membranes that expose Trp groups that favor cellular uptake [23]. Stability of siRNA-to-serum exposure improved as CADY molar ratio increased, indicating that CADY protects the siRNA within nanoparticle complexes. Potency of these complexes was confirmed in vitro in difficult to transfect cell lines, however no in vivo data has been reported in the literature to date. One of the potential limitations of in vivo development of CPPs is the potential for degradation by intracellular and extracellular proteases. To address this, CADY-K peptide was modified into a retro-inverso peptide, whereby peptides consist of D-amino acids in the reverse sequence of naturally occurring L-isoforms [59]. This peptide, termed RICK (Retro-Inverso CADY-K) had similar siRNA-mediated knockdown efficacy to parent CADY-K nanoparticles but with the advantage of improved resistance to enzymatic and serum degradation of siRNA cargo [24]. Another approach to CPP-mediated siRNA delivery has been the development of branched histidine-lysine (HK) polymers [60], whereby the histidine component is required for buffering and lysing of endosomes for cellular release of cargo and the lysine component important for electrostatic binding to nucleic acid. Utilizing this noncovalent approach, HK polymers containing siRNA directed against Raf-1, which has an important role in tumor angiogenesis, was able to effectively reduce xenograft tumor size by approximately 50% following multiple systemic dosing of 50 µg of HK/Raf-1 siRNA complexes during tumor growth, with no overt toxicity detected [32].

Hydrophobicity can also be conferred to CPPs through the addition of stearic acid modifications. One of the more widely published and successful examples is the PepFect peptide family. These peptides were initially developed through addition of a N-terminal stearic acid modification to transportan 10 (TP10), termed Pepfect 3, which enhanced splice-switching activity of 2′-OMe ASOs approximately 30-fold compared to unmodified peptide in a luciferase based cell assay [25]. Surprisingly, this peptide was inactive for RNAi-mediated silencing and so to address this, an endosomotropic modification with a chemical analogue of chloroquine was introduced to enhance endosomal release (Pepfect6) [26]. As well as exhibiting enhanced siRNA potency over commercially available reagents in vitro, Pepfect6 siRNA complexes induced potent target gene knockdown of 60–75% in kidney, lung, and liver organs following a single dose of 1 mg/kg with no apparent acute toxicity. Further iterations of this peptide through modification of the TP10 sequence (PepFect14) were also effective for multiple nucleic acid cargoes in vitro [27,28] and most interestingly could demonstrate that it could retain activity after being dried as a solid formulation for several months [28]. Perhaps surprisingly, these lipid-functionalized CPPS (PepFect and others) could be noncovalently formulated with otherwise difficult to transfect charge-neutral PMOs so as to induce effective splice-switching activity in vitro, although this could not be replicated for in vivo delivery [61].

A number of approaches to CPP design have been made to improve the selectivity of cell-type specific delivery of nucleic acid cargoes, especially for siRNA. Monoclonal antibodies or their fragments, aptamers, homing peptides, and small molecules can be employed to improve cell or tissue specific targeting and are generally conjugated or fused with cationic CPPs to improve interaction with anionic siRNA. Conjugation of CD7-specific single chain antibody to oligo-9-arginine CPP was used for highly efficacious siRNA delivery into primary human CD3^+^ T cells, with proof of targeting demonstrated in mice protected from HIV challenge following systemic administration of CCR5-, *Vif*-, and *Tat*-targeting siRNA nanoparticles to prevent viral spread and replication [62]. A short peptide derived from the rabies virus glycoprotein (RVG), which is known to specifically bind to acetylcholine receptors in neuronal cells, was functionalized for siRNA complexation through addition of nine arginine residues (RVG-9R) [29]. Intravenous administration of 50 µg of RVG-9R siRNA complexes into wild-type mice was able to induce approximately 50% reduction in Cu-Zn superoxide dismutase (*SOD1)* levels in the brain but not in spleen and liver, with efficacy peaking at 48 hours before returning to normal levels at around 9 days. Additionally, this approach was used in a mouse model of fatal flaviviral encephalitis with 80% survival in RVG-9R antiviral siRNA treated mice compared to control peptide-siRNA groups who all died within 10 days [29]. An alternate to cell-specific targeting is to design activatable CPPs (ACPP) that improve uptake under specific environmental conditions. These CPPs are generally hairpin structures that consist of a cationic and neutralizing anionic domain connected by a cleavable peptide loop that have limited cellular uptake due to neutralization of electrostatic interactions with the cell. However, in the presence of a disease-associated protease, the linker loop is cleaved, thus activating the cationic domain of the CPP [63]. This approach has been demonstrated for targeting of cargo-free CPPs to xenograft tumor models from different cancer sites where uptake is mediated by matrix metalloproteinase cleaveage of the CPP linker at the tumor-stromal interface [64]. Another reported ACPP strategy is to create a pH-sensitive linkage, such as hydrazine, that will be catalyzed under decreased pH conditions such as that observed in tumor tissues. Addition of a pH-sensitive ACPP to a liposomal siRNA carrier improved uptake and target-gene silencing in vitro under reduced pH conditions [65], although more optimization is required as the potency was not as high the original CPP and this approach has yet to be demonstrated in vivo to our knowledge. Sensitivity to pH can also be exploited by fusogenic peptides that can be functionally activated through conformational changes at low pH such as in acidic endosome and lysosomes that aid in cargo release from these compartments. An early example of this is the HA2 peptide derived from hemagglutinin which has a random coil structure at physiological pH, but adopts a α-helical conformation within the acidic endosome that fuses and destabilizes the endosomal membrane [66]. More recently, a fusion peptide, termed 599, consisting of a synthetic influenza virus-derived fusogenic domain and a cationic arginine domain, could induce significant target-gene silencing and subsequent decrease in cell invasiveness in an oral cancer cell model [30]. Tissue-specific targeting capabilities of this peptide was further enhanced through combining with an epidermal growth factor receptor targeting peptide and utilized for in vivo administration to xenograft oral cancer tumors, where 49% gene knockdown could be detected with the dual peptide nanoparticles but only 23% with the 599-siRNA alone [31].

## 4. Mechanism of CPP Mediated Uptake

**Figure 2:**
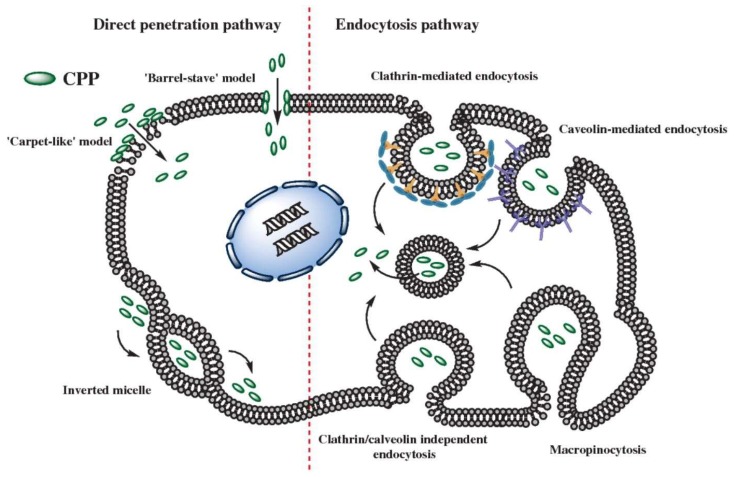
Cell-penetrating peptide internalization occurs through direct penetration (**left**) or endocytic pathways (**right**). Direct translocation of CPPs into the cellular space can occur through energy-independent mechanisms that cause membrane destabilization such as the “carpet-like” model or formation of inverted micelles, or through direct pore formation such as the “barrel-stave” model. The majority of CPPs are thought to be taken up through energy-dependent endocytic internalization either through clathrin-dependent, caveolin-mediated, and clathrin/caveolae independent endocytosis or micropinocytosis before escape from endosome compartments into the cellular environment.

The mechanism of cellular internalization of CPP, with or without cargo, can be broadly defined as either through energy-independent direct penetration or through energy-dependent endocytosis (graphically represented in Figure 2).The observation in early CPP experiments that uptake was similar at 4 °C and 37 °C, or under energy depletion conditions, suggested that direct translocation of the negatively-charged plasma membrane occurred via an energy-independent mechanism [6,67]. Direct translocation is thought to occur primarily through membrane destabilization or transient pore formation. In the “carpet-like” model, accumulation of lytic CPPs at the cell surface, primarily through electrostatic interaction, forms a localized “carpet”, leading to reorganization of the lipid membrane such that transient holes are formed that allow additional CPPs to enter [68]. In contrast, the “barrel-pore” model describes the formation of transmembrane channels that are formed due to insertion of amphipathic α-helices peptides whereby the hydrophobic surface interacts with the lipid membrane, whilst the hydrophilic surface faces inwards, thus forming an aqueous pore [69]. An alternate proposed model for direct uptake is the “inverted micelle model”, whereby CPP interaction with the lipid bilayer would result in the formation of inverted micelles that would trap the peptides in the hydrophilic core until further interaction with the membrane would cause the inverse process and release CPPs into the intracellular compartment [70]. However, a key subsequent study suggested that some of these early observations of direct penetration may indeed have been an artifact of cell fixation methods [71], and as a consequence, the majority of microscopic studies on CPP localization are conducted in live cells. Nevertheless, direct translocation cannot be entirely ruled out as, for example, CADY CPPs complexed with siRNA do not colocalize with endosomal markers and were active in the presence in of endocytic inhibitors [72]. Since these early studies, a number of energy dependent endocytic pathways including micropinocytosis [73], clathrin- [74], and caveolae-mediated [75] endocytosis have since been identified to be the primary driver of some CPPs, with and without nucleic acid cargoes (reviewed here [76]). In a comparison of several cationic and amphipathic CPPs conjugated to PNA, results suggested that cationic conjugates relied on micropinocytosis, whilst amphipathic conjugates utilized clathrin-mediated endocytosis [77]. However, endocytic pathways for each CPP class may not be very clearly defined as a large body of information suggests that the specific endocytic pathways utilized by CPPs is highly dependent on properties of the cargo, relative concentration, and the cell line/tissues being targeted [3]. A clear example of this is for Pip6A-PMO, whereby caveolae-mediated endocytosis is primarily responsible for uptake in skeletal muscle cells, but clathrin-mediated endocytosis was important for cardiomyocyte uptake [78]. More recently, cell-surface proteoglycan (glycosaminoglycans like heparan sulphates) [79] and other cofactors such as scavenger receptors (SR) have also been implied in the uptake mechanism of CPP nanoparticles [80]. SRs are a large family of pattern recognition receptors that are highly expressed in immune cells and play important roles in innate immunity and homeostasis [81]. Negatively charged Pepfect 14-SSO complexes were taken up into HeLa cells through a class A SR-dependent endocytosis pathway [82] and in subsequent studies, it was also demonstrated that some amphipathic and cationic CPPs could also trigger recruitment of SR-A3 and SR-A5 to the plasma membrane [83]. SRs have also been implicated in the uptake of spontaneously forming amphipathic CPP-PMO micelles, with SR knockout models having significantly reduced splice-switching activity in diaphragm and heart tissues compared to wild-type controls [84]. In an elegant study to further understand and identify uptake mechanisms of CPPs, RNA expression profiling of the early cellular response was performed in HeLa cells transfected with PepFect14 with or without an oligonucleotide cargo [85]. IPA analysis suggested that the autophagy pathway was being induced upon transfection and to validate this, ligands that modulate autophagy were coadministered with PF14-SSO in a HeLa luciferase splice-switching reporter system. Modulation of autophagy-related intracellular pathways showed concentration dependent effects on splice correction activity, and furthermore, autophagy induction and colocalization with autophagosomes was confirmed by confocal microscopy and transmission electron microscopy.

Endocytic uptake of CPPs and their cargo is followed by complex intracellular trafficking towards early endosomes, maturation in late endosomes/multivesicular bodies (MVBs), lysomes, or the Golgi network [86]. As one of the key limiting factors for the bioavailability of CPP cargo that enter the cell through these pathways is endosomal entrapment, researchers are actively pursuing bioengineering methods such as fusogenic lipids [87] for early escape from endosomes to the cytosol to prevent destruction and thus aid in the delivery to active sites in the cytoplasm [88]. A number of approaches have focused on taking advantage of the low pH environment of endosomes. The incorporation of pH-sensitive domains into the CPP has been used to destabilize lipid membranes under the acidic pH conditions in the endosome to aid escape [89]. Similarly, histidine moieties, which act as a proton sponge at endosomal pH, can increase osmotic pressure leading to endosomal rupture and cargo release [90]. Incorporation of lysomotropic moieties have also been used to successfully improve endosomal escape, as exemplified by Pepfect 6, that incorporated four chloroquine analogs to improve efficiency of cargo delivery compared to the parent stearyl-TP10 peptide [26].

## 5. Future Perspectives of Clinical Application of CPP-ASOs

Whilst the field of CPP-mediated delivery of oligonucleotides has expanded rapidly within the last 10 years, there have been no successful clinical trials to date that have utilized these approaches. To a large extent, this is a reflection as to where the field is at the moment, with studies focused on optimization of CPP design and generation of proofs of principle in early preclinical studies. Improvement in CPP design for covalent and noncovalent applications of CPP are focused predominantly on improving cellular uptake, although tissue-specific targeting is an obvious goal. There are approximately 1700 unique CPPs currently listed on the CPPsite 2.0 database [91] that have been experimentally validated, and as such, represent expensive and time-consuming studies. To attempt to improve upon CPP prediction, differences between true and non-CPPs have formed the basis for various machine learning models to predict whether novel peptide sequences are cell-penetrating or not. A recent example of this includes a web-based server that also predicts uptake efficiency of candidate CPP sequences [92]. Although experimental validation will always be necessary, these algorithms may improve the hit-rate and broaden the design landscape further outside of the major CPP families.

There are a number of advantages and disadvantages to developing covalent and noncovalent approaches for clinical application of CPP-ASO delivery. For covalent conjugations, the advantages are that a clearly defined entity is produced that has high reproducibility and generally has a low molar ratio of CPP-to-ASO cargo (typically 1:1), which is important in consideration of CPP toxicity. However, production of these compounds is quite laborious and limited predominantly to charge-neutral oligonucleotide backbones, which in turn have more limited biological application, being used primarily for steric-blocking approaches such as exon-skipping. The process of noncovalent complexation due to electrostatic and hydrophobic interactions between CPPs and ASOs is more simplified, usually by coincubation at defined concentrations. There is also the advantage that this approach can be used for a wider range of nucleic acid chemistries as well as types of effector molecules and biological applications such as RNA interference and plasmid DNA delivery. However, with this simplified complexation process comes the drawback of heterogeneity of the nanoparticle-like complexes that are formed and the potential for aggregation. As size, shape, and charge of the nanoparticle are crucial for uptake efficacy, uniform production is desirable to be considered for clinical application. Assessment of size and morphology of Pepfect and Nickfect nucleic acid nanoparticles by transmission electron microscopy demonstrated that a relatively homogenous population of nanoparticles ranging in size from 30 to 60 nm were produced in the absence of aggregation, suggesting that, at least for these peptides, relative homogeneity could be achieved [93]. CPP mediated toxicity is also a concern for both approaches and for noncovalent approaches, especially where typically a higher molar ratio of CPP:ASO from 5:1 to 20:1 is required for optimal efficacy [22,23,26,28].

For clinical development, safety, tolerability, pharmacokinetics, pharmacodynamics, and therapeutic index are major factors to be considered for nucleic acid delivery. Unfortunately, in vivo assessment of CPP-ASO toxicology has not been robustly reported, although assessment of serum markers of liver damage at efficacious dosing for CPP-PMO has not indicated any hepatic toxicity, at least for arginine-based CPPs [14]. One of the potential advantages of CPP-based approaches over viral-based delivery is the opportunity for readministration which is not possible for viral vectors which elicit a strong immune response upon subsequent administration or if patients have pre-existing viral exposure. In multiple preclinical studies, CPP-ASOs toleration of multiple-dosing regimens is reported, and for Pepfect 6 mediated delivery of siRNA, no acute inflammatory cytokine response could be detected and no increases in serum markers of kidney or liver damage were observed following intravenous delivery [26]. Furthermore, addition of Tat, Antennapedia, and Transportan CPPs to epithelial cells at concentrations effective for cargo uptake, did not elicit toll-like receptor signaling or inflammatory cytokines [94]. In the clearest insight of CPP-ASO toxicity, administration of high dose B-peptide-PMO into rats demonstrated a decrease in body weight and elevated serum blood urea nitrogen and creatinine in a dose-dependent manner that suggested reduced renal output [40]. Administration at very high doses (>150 mg/kg) were also accompanied with mild lethargy immediately post-administration. However, it should be noted that these responses were observed at doses significantly higher than minimal effective doses. In a clinical development program by AVI-BioPharma (now Sarepta Therapeutics), nonhuman primates were administered once-weekly IV administration for 4 weeks of 9 mg/kg of (RXRRBR)_2_ peptide conjugated PMO that demonstrated an average of 40%, 25%, and 2% exon skipping in diaphragm, quadriceps, and heart, respectively [95]. However, at this dose, mild tubular degeneration was detected in the kidneys and development of this compound has been dropped. However, recent investor presentations from Sarepta have shown data from nonhuman primate studies of a novel CPP-PMO (SRP-5051) targeting exon 51 of *DMD* that is well tolerated and shows efficacious exon-skipping activity. This has led to acceptance of an Investigational New Drug application and a phase 1/2a trial is currently underway and estimated to be completed early 2019 (NCT03375255 ClinicalTrials.gov)

The field of CPP delivery of oligonucleotides clearly shows promise as evidenced by the number of successful preclinical studies in a wide range of disease indications that have arisen over the last decade. The greatest success is likely to come for targeting organs that are most refractory to systemic naked oligonucleotide delivery such as the central nervous system, heart, and skeletal muscle where the need for effective delivery is paramount to realize the power of specific gene and disease mutation targeting that oligonucleotides offer. With advancement of design algorithms and better understanding of uptake mechanisms and intracellular trafficking, the therapeutic potential of this field will improve and likely we will see more CPP-ASO candidate’s move towards clinical drug development.

## Figures and Tables

**Table 1:** Examples of cell-penetrating peptide sequences and nucleic acid cargo application described in this review. SSO, splice-switching oligonucleotide; DMD, Duchenne muscular dystrophy; DM1, Myotonic dystrophy type I; SMA, Spinal muscular atrophy; 2OMe, 2′-*O*-methyl.

Peptide	Sequence	Application
Covalent conjugated CPPs		
B	(RXRRBR)_2_XB	SSO for DMD, DM1 [9,10,11]
B-MSP	(RXRRBR)_2_XBASSLNIA	SSO for DMD [12]
Pip6	RXRRBRRXR YQFLI RXRBRXRB	SSO for DMD, SMA [13,14,15,16]
M12	RRQPPRSISSHP	SSO for DMD [17]
Br-ApoE(K→A)	Ac-LRALRARLLRGGAc-LRALRARLLRGGKX-Bpg-G	SSO for SMA [18]
P4	LGAQSNF	SSO (2OMe) for DMD [19]
(RXR)_4_	RXRRXRRXRRXR	anti-viral anti-bacterial [20,21]
Nanoparticle forming CPPs		
MPG-8	βAFLGWLGAWGTMGWSPKKKRK-Cya	siRNA for xenograft tumor model [22]
CADY	Ac-GLWRALWRLLRSLWRLLWRA-Cya	siRNA, cell lines (various) [23]
RICK	KWLLRWLSRLLRWLARWLG	siRNA, human glioblastoma cells [24]
Pepfect 3	stearyl-AGYLLGKINLKALAALAKKIL-NH_2_	Plasmid DNA, cell lines, intramuscular [25]
Pepfect 6	See reference	siRNA, cell lines (various), systemic IV [26]
Pepfect 14	stearyl-AGYLLGKLLOOLAAAALOOLL-NH_2_	Plasmid DNA, SSO [27,28]
RVG-9R	YTIWMPENPRPGTPCDIFTNSRGKRASNGGGGRRRRRRRRR	siRNA, brain-targeting disease models [29]
599	GLFEAIEGFIENGWEGMIDGWYGGGGRRRRRRRRRK	siRNA, oral cancer [30,31]
H3K(+H)4b	Branched KHHHKHHHKHHHHKHHHK	siRNA, tumor xenograft [32]
